# The Role of Income in System Justification

**DOI:** 10.11621/pir.2023.0104

**Published:** 2023-03-30

**Authors:** Elena R. Agadullina

**Affiliations:** a HSE University, Moscow, Russia

**Keywords:** System justification, income, life satisfaction, control over life

## Abstract

**Background:**

System justification theory asserts that people’s motivation to defend, justify, and maintain the status quo depends on their socio-economic status. At the same time practically nothing is known about the mediators for the relationship between a person’s income and his adherence to system justification.

**Objective:**

The aim of this study was to clarify the role of income in motivating an individual to justify the system, taking into account as potential mediators of this relationship his sense of control over life and level of life satisfaction.

**Design:**

In an online study (N = 410), a double sequential mediation model was tested, with an individual’s income as an independent variable, his/her system justification as a dependent variable, and his/her perceived control over life and level of life satisfaction as mediators. The impact of education was controlled by inserting it into the model as a covariate.

**Results:**

The results demonstrated that people with low incomes justify the system more than people with high incomes do. At the same time, there was a positive indirect effect of income on system justification, indicating that, compared to people with low incomes, those with high incomes had a more pronounced sense of control over their lives, which contributed to an increase in their level of life satisfaction, and was positively associated with justification of the status quo.

**Conclusion:**

The results are discussed in terms of differences in the palliative function of system justification for individuals of different socio-economic status.

## Introduction

System justification (SJ) is a process of rationalizing the existing social, economic, and political relationships between people with different social statuses as essential, legitimate, and fair (Jost & Banaji, 1994). One of the basic assumptions of SJ theory is that people of low status are more motivated to defend, justify, and maintain the status quo than those of higher status (the status-legitimacy hypothesis). This hypothesis describes a paradoxical assumption, according to which inequality in society is mainly supported by groups that suffer most from it. This assertion has become one of the most discussed theses of SJ theory.

Yet empirical testing of this status-legitimacy hypothesis has not confirmed it as far as subjective socio-economic status (SES) (perceived position in society in comparison with other social groups [see, for example, Adler et al., 2000]) is concerned. In particular, in various cross-cultural studies, subjective SES was positively associated with different elements of SJ: *i.e*., trust in government and social institutions (Brandt, 2013; Brandt et al., 2020); perception of social justice (Caricati et al., 2012) and income distribution (Caricati, 2017); perception of the legitimacy of the status quo (Brandt et al., 2020); and the scores on the SJ scales (Vargas-Salfate, Paez, Khan, et al., 2018; Vargas-Salfate, Paez, Liu, et al., 2018).

The positive relationship between subjective SES and SJ can be explained by the fact that people often cannot adequately assess their social status; they tend to place themselves in the middle of the social hierarchy (see, for example, Evans & Kelley, 2004). As a result, people with objectively low status often overestimate their social position, which allows them to maintain a positive self-representation and self-esteem, and leads them to defend their overvalued position, including by justifying the existing system (Brandt et al., 2020).

As for the objective SES (education level and income) (Kraus et al., 2011), there are much less data and more ambiguous findings. When objective SES was operationalized by education, it was almost always negatively associated with various forms of SJ (Brandt, 2013; Li, Yang, et al., 2020). This is because the level of conservatism mediates the relationship between education and SJ: less-educated people are more conservative (Jost et al., 2017; Li, Yang, et al., 2020), and conservatism predicts SJ (Vargas-Salfate, Paez, Liu, et al., 2018).

The situation regarding the effect of income on SJ is more complicated. Li with colleagues (2020) showed that income had either a significant negative (Studies 1–3) or non-significant correlation with SJ (Studies 4–5). Vargas-Salfate with colleagues (2018), using cross-cultural data, found that income was negatively associated with willingness to justify the system, while Brandt (2013) demonstrated that for different countries, income was not associated with trust in government and public institutions. These conflicting results can reflect a variety of reasons, such as the mixing of the effects of education and income on SJ, since education is often a precondition for well-paid and high-status jobs, or existing indirect effects that determine the relationship between income and SJ. The main aim of this study was to clarify the role of income in determining SJ.

## Mediators for the relationship between income and system justification

According to the basic assumption of SJ theory, people with a low social status are more likely to justify the system because they feel cognitive dissonance between the need to maintain a positive representation about themselves and their ingroup, and the recognition that the status quo assigns them a low social status (Jost & Banaji, 1994). The need to resolve this dissonance is realized, among other things, through SJ. However, while it has been shown that the level of conservatism explains the link between education and SJ, there are no data about mediators for the relationship between income and SJ. At the same time, previous studies on how income is related to different psychological outcomes (Manstead, 2018). Pitlik and Rode (2016) showed that income is linked with perceived control over life: the lower the income, the fewer alternatives individuals have to choose from; therefore, their sense of control over life decreases.

Kraus and his colleagues (2012) summarized the existing findings on the differences between people with various income levels and found that low-income individuals were more motivated to cope with current threats and make situational attributions, while individuals with a high income were more focused on achieving their goals and favored dispositional attributions. For its part, a sense of control over life also increases a person’s willingness to achieve goals, which, in turn, contributes to maintaining high social status and material well-being (Johnson & Krueger, 2006). Moreover, a sense of control over life allows individuals to maintain subjective wellbeing and significantly enhances their levels of happiness and life satisfaction (Inglehart et al., 2008; Klonowicz, 2001; Myers & Diener, 1995).

Income also makes a significant contribution to life satisfaction (Boyce et al., 2010; Graafland & Lous, 2018; Salinas-Jiménez et al., 2010). Howell and Howell (2008) showed in their meta-analysis that life satisfaction increases with income, as higher income creates more opportunities to meet basic physiological and psychological needs (*e.g.,* good nutrition, safety, and so on), while low income is often accompanied by stress due to the difficulty or inability to meet daily needs. Thus, income, both directly and indirectly via perceived control over life, impacts life satisfaction.

For its part, life satisfaction is associated with SJ. According to SJ theory, justification of the status quo fulfills a palliative function as it enhances positive, and reduces negative, experiences (Jost & Hunyady, 2005). A comparative study conducted in 18 countries confirmed that SJ was positively associated with life satisfaction and negatively with anxiety and depression, regardless of the person’s social status and the level of inequality in society (Vargas-Salfate, Paez, Khan, et al., 2018). Li with colleagues (2020), based on data from China, also confirmed that SJ was positively associated with life satisfaction, regardless of the social class to which the individuals belong.

Taken altogether, these results enable us to conclude that the relationship between income and SJ can be mediated by psychological attitudes that are associated with high or low income. Thus, the main research question of this study: How do different psychological variables mediate the relationship between income and SJ? The answer to this question was sought through testing the hypothesis: High income increases an individual’s sense of control, which increases his or her life satisfaction, which, in turn, increases SJ.

## Methods

### Participants

The sample consisted of 410 Russians (66.3 % women, M_age_ = 38.02, *SD* = 10.53) who were recruited for an online study and received a participation fee. Most of the respondents were native Russians (92.9%); 48.8% were non-believers and 37.1% were Orthodox; 50.5% had completed higher education; and 37.6% lived in cities with a population of more than a million, including Moscow and St. Petersburg.

### Procedure and Measures

The respondents filled out an informed consent form, after which they were introduced to the purpose of the study and provided with instructions. They next completed the questionnaires about life satisfaction, perceived control over life, and SJ, and then answered the social-demographic questions.

*Life satisfaction* was measured using ratings from the World Values Survey (WVS): “How satisfied are you with your life in general?” The respondents had to rate their degree of satisfaction on a scale from 0 (*completely dissatisfied*) to 10 (*completely satisfied*).

*Control over life* was also measured using WVS ratings: “Some people feel they have completely free choice and control over their lives, while other people feel that what they do has no real effect on what happens to them. To what extent do you control the course of your life?” Respondents had to rate how much, in their opinion, they had freedom of choice and control over their lives, on a scale from 1 (*I have absolutely no control over my life and what happens in it*) to 7 (*I am completely in control of my life and what happens in it*).

*System justification* was measured by the Russian version of the system justification scale (Agadullina et al., 2021), which included five statements (*e.g.,* “In Russia today, life does not need any significant changes”) (a = 0.91). The level of agreement with the statement was assessed on a scale from 1 *(strongly disagree*) to 9 (*strongly agree*).

*Income* was measured by the statement: “Please, indicate your income per month.” Respondents had to choose one of 14 options: 1 = *less than 15,000 rubles per month*; 2 = f*rom 15,000 to 20,000 rubles per month*; 3 = *from 20,000 to 30,000 rubles per month*; and so on up to 14 (*more than 200,000 rubles per month*).

*Education* was measured by asking the respondents to state their highest level of education from the options: 1) primary, 2) secondary (school), 3) secondary special (college), 4) incomplete higher education, 5) completed higher education, and 6) two or more higher education degrees.

### Data analysis

A double sequential mediation model was used for data analyses in the PROCESS v 3.3 (model 6) for SPSS (Hayes & Preacher, 2014). An individual’s income level was used as an independent variable, SJ as a dependent variable, and perceived control over life and the level of life satisfaction as the mediators. The effect of education was controlled by inserting it into the model as a covariate. The indirect effect was tested using a bootstrapping procedure (10,000 random samples). If the 95% bootstrapping confidence interval did not include zero, then the effect was considered significant (MacKinnon et al., 2004).

## Results

### Descriptive statistics

*Table 1* shows the descriptive statistics and correlations between the variables. In full accordance with previous findings, income positively correlated with perceived control over life and life satisfaction; at the same time, these variables correlated with each other and with SJ.

**Table 1:** Descriptive statistics and intercorrelations for variables.

	M	*SD*	Min	Max	Skewness	Kurtosis	1	2	3	4
1. **Life satisfaction**	5.51	2.19	1	10	–.135	–.782	–			
2. **System justification**	3.88	1.87	1	9	.233	–.671	.494**	–		
3. **Income**	3.33	2.10	1	14	1.397	2.993	.256**	.120*	–	
4. **Control over life**	4.76	1.33	1	7	–.147	–.347	.561**	.382**	.153**	–
5. **Education**	4.25	1.24	2	6	–.050	–.439	.090	–.023*	.201**	–.018

Note. ** p < .01, * p < .05

Education was not correlated with life satisfaction and a sense of control over life (the higher the level of education, the less perceived control over life respondents felt). Despite the significant positive correlation between income and education, these variables showed different patterns in relation to SJ. So, SJ was positively associated with income and negatively associated with education.

### Double sequential mediation model

The results presented in *Table 2* demonstrate that income had a positive effect on control over life [*B* = .10, *t* (409) = 3.26, *p* = .001] and life satisfaction [*B* = .17, *t* (409) = 3.88, *p* < .001]. At the same time, income had a negative effect on system justification [*B* = –.01, *t* (409) = –.06, *p* = .042], demonstrating that people with low income justify the system more than those with high income, which can be considered as a confirmation of the status legitimation hypothesis. The indirect effect of income on SJ via life satisfaction [B = .06, 95% CI (.03, .10)], as well as via control over life [B = .02, 95% CI (.01, .04)], was significant, indicating that people with a high income were more satisfied with life and felt more control over life than those with low income. The results of double sequential mediation via control over life and life satisfaction were also significant [B = .03, 95% CI (.01, .06)].

**Table 2:** The results of the double sequential mediation model.

	Control over life		Life satisfaction		System justification	
	Beta (*SE*)	95 % CI	Beta (*SE*)	95 % CI	Beta (*SE*)	95 % CI
Income	.10** (.03)	.04, .17	.17*** (.04)	.08,.25	–.01* (.01)	–.08, –.01
Control over life			.89*** (.07)	.75, 1.02	.21** (.07)	.06, .35
Life satisfaction					.35*** (.05)	.27, .44
Education	–.05 (.05)	–.15, .05	.12 (.07)	–.02, .26	–.09* (.07)	–.21, –.04
Constant	4.64*** (.24)	4.17, 5.11	0.23 (.45)	–.65, 1.11	1. 31** (.41)	.51, 2.11
	*R*^2^ = .03		*R*^2^ = .35		*R*^2^ = .26	
**Total effect income on system justification Indirect effect**					.11** (.04)	.03, .20
Income — control over life — system justification					.02 (.01)	.01, .04
Income — life satisfaction — system justification					.06 (.02)	.03, .10
Income — control over life — life satisfaction — system justification					.03 (.01)	.01, .06

Note. * p < .05, ** p < .01, *** p < .001

In general, these results confirmed that people with high incomes felt more control over life compared to those with low incomes. This increased their life satisfaction, which, in turn, increased their willingness to justify the status quo. Thus, the direct and indirect effects of income on system justification showed opposite outcomes. Moreover, psychological variables associated with income completely mediated its relationship with SJ; the total effect was significant [*B* =.11, *t* (409) = 2.59, *p* = .01].

The level of education had the expected negative significant direct effect on SJ [*B* = –.09, *t* (409) = –1.34, *p* = .034], but the total effect of education on SJ was non-significant [*B* = –.07, *t* (409) = –.98, *p* = .328].

## Discussion

The aim of this study was to clarify the role of income in SJ and to investigate the relationship between income and SJ, taking into account as potential mediators perceived control over life and the level of life satisfaction.

The results confirmed that the status legitimacy hypothesis is supported when the relationship between objective income and SJ is studied, regardless of the psychological variables associated with high or low income. Following the assumption of SJ theory (Jost & Banaji, 1994), and previously obtained cross-cultural results (Vargas-Salfate et al., 2018), it was found that low-income individuals justified the status quo more than high-income individuals. The main hypothesis of this study, that the relationship between income and SJ can be mediated by psychological variables associated with high and low income, has also been confirmed.

In previous studies, it has been shown that higher income increased feelings of control over life (Kraus, Piff, Mendoza-Denton, et al., 2012; Pitlik & Rode, 2016) and life satisfaction (Boyce et al., 2010; Graafland & Lous, 2018; Salinas-Jiménez et al., 2010). For their part, both the degree of control over life and life satisfaction contribute to strengthening SJ (Jost & Hunyady, 2005; Vargas-Salfate, Paez, Khan, et al., 2018). Thus, this study reproduced the main results obtained in previous studies. The main new contribution of our results was that they demonstrated that including the psychological mediators in the analysis of the relationship between income and SJ completely changes the results, since they contribute to the fact that people with high incomes begin to justify the system more than those with low incomes.

These results highlight the differences that the palliative function of SJ can have for groups of different status. The direct negative effect of income on SJ indicates that system-justifying ideology may help people with low status cope with negative emotions related to their low social position and their inability to fulfill the important physiological and psychological needs (Vargas-Salfate, Paez, Khan, et al., 2018). However, for low-status groups this palliative effect on subjective well-being is short-term; in the long term, low social status leads to a significant decrease in an individual’s subjective well-being, and SJ is not able to neutralize its negative consequences (Jost & Hunyady, 2003). By contrast, for high-status groups, SJ is positively and strongly associated with well-being in both the short and long term. In other words, the rich become richer, since subjective well-being is added to high material well-being, while poor people can increase their well-being in the short term by justifying the system, but in the long term, they suffer from the system and increasingly justify it less.

In a broader context, the results obtained raise several questions, especially relating to the debate about subjective and objective measures in psychology. Obviously, one should separate subjective and objective status when it comes to testing the status legitimacy hypothesis, since when social status is operationalized through income or education, this hypothesis is more likely to be confirmed. Li with colleagues (2020) suggested that many of the conflicting results related to the reasons for justifying the status quo may be precisely associated with differences in the operationalization of perceived status. Undoubtedly, further reflection on these differences is required within the framework of SJ theory.

Moreover, even when it comes to objective status, the effects of income and education must be considered separately. In the present study, education had a negative effect on SJ, which confirms previous findings (Jost et al., 2017; Li, Yang, et al., 2020). Previous studies have also found that both income and education can be negatively associated with different system-justifying ideologies such as right-wing authoritarianism (RWA) and social dominance orientation (SDO) and prejudice, but the effect of education is stronger than the effect of income (Carvacho et al., 2013; Schiefer, 2013). This trend is explained by the fact that education is inextricably linked to the inculcation of democratic and egalitarian values (Hainmueller & Hiscox, 2007), the maintenance of which is less dependent on situational circumstances, while income can be highly dependent on different socio-economic conditions (for example, an economic crisis, high unemployment, low demand for certain professions, etc.).

## Limitations

The presented research has several limitations. First of all, the measurements used for control over life and life satisfaction are more typical of sociological research (only one item for each construct). This could have somewhat oversimplified the researched reality. Second, the study did not take into account other potential mediators (*e.g.,* conservatism). For example, Johnson and Krueger (2006) demonstrated that income satisfaction fully mediated the relationship between real income and life satisfaction. At the same time, the broader socio-cultural and socio-economical contexts are no less important. In countries with low economic development, an increase in income makes a large contribution to life satisfaction, primarily by increasing the ability to fulfill physiological needs. But in economically developed countries, this connection is less pronounced, since the needs for self-realization and various psychological needs begin to play a larger role than physiological ones (Oishi et al., 1999).

Russia, where this study was conducted, belongs to the group of countries with dominant survival values (in contrast to self-expression values) (World Values Survey, 2020) and is not a typical WEIRD (Western, Educated, Industrialized, Rich, and Democratic) country (Henrich et al., 2010). As a result, for Russians there may be a stronger relationship between income and life satisfaction, and hence a more pronounced indirect effect of income on SJ via life satisfaction than in other developed countries with other core values. In future studies, it would be productive to expand the variables included in the analysis. For the correct interpretation of this study’s results, it is necessary to take into account the described limitations.

## Conclusion

The results confirmed that the relationship between income and SJ is mediated by psychological variables associated with high and low income (perceived control over life and the level of life satisfaction). Undoubtedly, both the direct and indirect relationships between income, education, and SJ need to be studied further in different socio-economic and cultural contexts to draw deeper conclusions.

## References

[ref1] Adler, N., Adler, N.E., Epel, E S., Castellazzo, G., & Ickovics, J.R. (2000). Relationship of Subjective and Objective Social Status With Psychological and Physiological Functioning: Preliminary Data in Healthy White Women. Health Psychology, 19(6), 586–592. 10.1037/0278-6133.19.6.586 11129362

[ref2] Agadullina, E., Ivanov, A., & Sarieva, I. (2021). How Do Russians Perceive and Justify the Status Quo: Insights From Adapting the System Justification Scales. Frontiers in Psychology, 12(October), 1–12. 10.3389/fpsyg.2021.717838 PMC8566909 34744879

[ref3] Boyce, C J., Brown, G.D.A., & Moore, S.C. (2010). Money and Happiness: Rank of Income, Not Income, Affects Life Satisfaction. Psychological Science, 21(4), 471–475. 10.1177/0956797610362671 20424085

[ref4] Brandt, M.J. (2013). Do the Disadvantaged Legitimize the Social System? A Large-Scale Test of the Status — Legitimacy Hypothesis. Journal of Personality and Social Psychology, 104(5), 765–785. 10.1037/a0031751 23421361

[ref5] Brandt, M.J., Kuppens, T., Spears, R., Andrighetto, L., Autin, F., Babincak, P., … Zimmerman, J.L. (2020). Subjective Status and Perceived Legitimacy across Countries. European Journal of Social Psychology, ejsp.2694. 10.1002/ejsp.2694 PMC7507836 32999511

[ref6] Caricati, L. (2017). Testing the Status-Legitimacy Hypothesis: A Multilevel Modelling Approach to the Perception of Legitimacy in Income Distribution in 36 Nations. The Journal of Social Psychology, 5, 532–540. 10.1080/00224545.2016.1242472 27684703

[ref7] Caricati, L., & Lorenzi-cioldi, F. (2012). Does status matter? Testing hypotheses from strong form of System Justification Theory. Revue Internationale de Psychologie Sociale, 25(1), 67–95.

[ref8] Carvacho, H., Zick, A., Haye, A., González, R., Manzi, J., Kocik, C., & Bertl, M. (2013). On the relation between social class and prejudice: The roles of education, income, and ideological attitudes. European Journal of Social Psychology, 43(4), 272–285. 10.1002/ejsp.1961

[ref9] Evans, M.D., & Kelley, J. (2004). Subjective social location: Data from 21 nations. International Journal of Public Opinion Research, 16, 3–38. 10.1093/ijpor/16.1.3

[ref10] Graafland, J., & Lous, B. (2018). Economic Freedom, Income Inequality, and Life Satisfaction in OECD Countries. Journal of Happiness Studies, 19(7), 2071–2093. 10.1007/s10902-017-9905-7

[ref11] Hainmueller, J., & Hiscox, M.J. (2007). Educated preferences: Explaining attitudes toward immigration in Europe. International Organization, 61(2), 399–442. 10.1017/S0020818307070142

[ref12] Hayes, A.F., & Preacher, K J. (2014). Statistical mediation analysis with a multicategorical independent variable. British Journal of Mathematical and Statistical Psychology, 67(3), 451–470. 10.1111/bmsp.12028 24188158

[ref13] Henrich, J., Heine, S.J., & Norenzayan, A. (2010). The weirdest people in the world? Behavioral and Brain Sciences, 33(2–3), 61–83. 10.1017/S0140525X0999152X 20550733

[ref14] Howell, R.T., & Howell, C.J. (2008). The relation of economic status to subjective well-being in developing countries: A meta-analysis. Psychological Bulletin, 134(4), 536–560. 10.1037/0033-2909.134.4.536 18605819

[ref15] Inglehart, R., Foa, R., Peterson, C., & Welzel, C. (2008). Development, Freedom, and Rising Happiness A Global Perspective (1981–2007). Perspectives on Psychological Science, 3(4), 264–285. 10.1111/j.1745-6924.2008.00078.x 26158947

[ref16] Johnson, W., & Krueger, R.F. (2006). How Money Buys Happiness: Genetic and Environmental Processes Linking Finances and Life Satisfaction. Journal of Personality and Social Psychology, 90(4), 680–691. 10.1037/0022-3514.90.4.680 16649863

[ref17] Jost, J., & Hunyady, O. (2003). The psychology of system justification and the palliative function of ideology. European Review of Social Psychology, 13(1), 111–153. 10.1080/10463280240000046

[ref18] Jost, J.T., & Banaji, M.R. (1994). The role of stereotyping in system‐justification and the production of false consciousness. British Journal of Social Psychology, 33(1), 1–27. 10.1111/j.2044-8309.1994.tb01008.x

[ref19] Jost, J.T., & Hunyady, O. (2005). Antecedents and Consequences of System-Justifying Ideology. Current Directions in Psychological Science, 14(5), 260–265. 10.1111/j.0963-7214.2005.00377.x

[ref20] Jost, J.T., Langer, M., Badaan, V., Azevedo, F., Etchezahar, E., Ungaretti, J., & Hennes, E.P. (2017). Ideology and the limits of self-interest: System justification motivation and conservative advantages in mass politics. Translational Issues in Psychological Science, 3(3), e1–e26. 10.1037/tps0000127

[ref21] Klonowicz, T. (2001). Discontented people: Reactivity and locus of control as determinants of subjective well-being. European Journal of Personality, 15(June 2000), 29–47. 10.1002/per.387

[ref22] Kraus, M.W., Piff, P.K., & Keltner, D. (2011). Social class as culture: The convergence of resources and rank in the social realm. Current Directions in Psychological Science, 20(4), 246–250. 10.1177/0963721411414654

[ref23] Kraus, M.W., Piff, P.K., Mendoza-denton, R., Rheinschmidt, M.L., & Keltner, D. (2012). Social Class, Solipsism , and Contextualism : How the Rich Are Different From the Poor. Psychological Review, 119(3), 546–572. 10.1037/a0028756 22775498

[ref24] Li, W., Wu, J., & Kou, Y. (2020). System Justification Enhances Life Satisfaction of High- and Low-Status People in China. Social Psychological and Personality Science, 11(5), 588–596. 10.1177/1948550619866182

[ref25] Li, W., Yang, Y., Wu, J., & Kou, Y. (2020). Testing the Status-Legitimacy Hypothesis in China: Objective and Subjective Socioeconomic Status Divergently Predict System Justification. Personality and Social Psychology Bulletin, 46(7), 1044–1058. 10.1177/0146167219893997 31896317

[ref26] MacKinnon, D., Lockwood, C., & Williams, J. (2004). Confidence Limits for the Indirect Effect: Distribution of the Product and Resampling Methods. Multivariate Behavioral Research, 39(1), 99–128. 10.1207/s15327906mbr3901 20157642 PMC2821115

[ref27] Manstead, A.S.R. (2018). The psychology of social class: How socioeconomic status impacts thought, feelings, and behaviour. British Journal of Social Psychology, 57(2), 267–291. 10.1111/bjso.12251 29492984 PMC5901394

[ref28] Myers, D.G., & Diener, E. (1995). Who Is Happy? Psychological Science, 6(1), 10–19. 10.1111/j.1467-9280.1995.tb00298.x

[ref29] Oishi, S., Diener, E.F., Lucas, R.E., & Suh, E.M. (1999). Cross-cultural variations in predictors of life satisfaction: Perspectives from needs and values. Personality and Social Psychology Bulletin, 25(8), 980–990. 10.1177/01461672992511006

[ref30] Pitlik, H., & Rode, M. (2016). Free to choose? Economic freedom, relative income , and life control perceptions. International Journal of Wellbeing, 6(1), 81–100. 10.5502/ijw.v6i1.390

[ref31] Salinas-Jiménez, M. del M., Artés, J., & Salinas-Jiménez, J. (2010). Income, Motivation, and Satisfaction with Life: An Empirical Analysis. Journal of Happiness Studies, 11(6), 779–793. 10.1007/s10902-010-9185-y

[ref32] Schiefer, D. (2013). Cultural Values and Group-Related Attitudes: A Comparison of Individuals With and Without Migration Background Across 24 Countries. Journal of Cross-Cultural Psychology, 44(2), 245–262. 10.1177/0022022112444898

[ref33] Vargas-Salfate, S., Paez, D., Khan, S.S., Liu, J.H., & Gil de Zúñiga, H. (2018). System justification enhances well-being: A longitudinal analysis of the palliative function of system justification in 18 countries. British Journal of Social Psychology, 57(3), 567–590. 10.1111/bjso.12254 29577342

[ref34] Vargas-Salfate, S., Paez, D., Liu, J.H., Pratto, F., & Gil de Zúñiga, H. (2018). A Comparison of Social Dominance Theory and System Justification: The Role of Social Status in 19 Nations. Personality and Social Psychology Bulletin, 44(7), 1060–1076. 10.1177/0146167218757455 29544389

[ref35] World Values Suvey. (2020). Cultural map. Retrieved from https://www.worldvaluessurvey.org/WVSContents.jsp

